# Gangliar Disease of Joints

**Published:** 1884-11

**Authors:** 


### Gangliar Disease of Joints.

Four cases of this interesting disease are reported by Mr. Norton, of St. Mary's Hospital, England. After relating the cases and defining it as a particular form of destructive disease of joints, totally different from scrofulous disease and from rheumatic disease in its pathology, though resembling either in external appearance, and often in its symptoms, the author then presents its chief characteristics as follows:

1. It is a disease which seems to occur almost invariably in elderly persons, or at any rate from thirty-five years upwards, though it does occur from time to time in children.

2. It is always a truly destructive disease, and for a certainty terminates in ulceration of the bones, preferably the small bones in the cases of the wrist and tarsus; the bones being rarefied and softened and often in part absorbed, according to the length of time during which the disease has existed before the commencement of the caries.

3. It is a disease which I am inclined to believe never allows improvement, though it may remain dormant for an indefinite period.

4. The synovial membrane is villous or granular, but never develops a mass of granulation tissue, such as usually occurs in the chronic destructive arthritis of so-called strumous children, and which has been called spongy synovial membrane.

5. It is a disease, of necessity, associated with the formation of ganglions, either connected with the joint, or at a distance from the joint, and then not communicating.

6. It is a chronic disease, extending often over ten to fifteen years, or perhaps more.

7. It is not painful to the touch, is not accompanied by much pain, though there is often aching, and the patient then refuses to use the limb; and, after a time the ligaments give way, and the hand (if wrist joint) hangs and cannot be lifted. When ulceration takes place with suppuration, then, of course, pain comes on, and the temperature rises.

8. When ulceration commences it progresses very rapidly, all the bones being affected, and some completely disappearing.

9. It appears to arise spontaneously, and not to result from injury.

The presence of ganglions will diagnose this disease from common ulceration of the cartilages or from rheumatic disease. In chronic synovitis the swelling and protrusion of the joint give a sense of fluctuation to the touch; in this disease the swelling is solid and not fluid. In differentiating from rheumatic disease, enlarged bursæ should not be mistaken for ganglionic formations.

It is a progressive disease and does not yield readily to treatment. The ganglions should never be opened, but they may be incised subcutaneously or the fluid aspirated. The fluid from the joint may also be aspirated, the limb placed upon a splint and a lotion of one part of tincture of iodine, four parts of glycerine, and sixteen parts of water, kept constantly applied. If the hand hangs or the joint glides there is no other course but amputation.—*British Medical Journal, Aug. 30, 1884.*

